# Practice Patterns and Outcomes Among Surgical Oncology Fellowship Graduates Performing Complex Cancer Surgery in the United States Across Different Career Stages

**DOI:** 10.1245/s10434-024-15436-0

**Published:** 2024-05-18

**Authors:** Diamantis I. Tsilimigras, Odysseas Chatzipanagiotou, Selamawit Woldesenbet, Yutaka Endo, Abdullah Altaf, Erryk Katayama, Timothy M. Pawlik

**Affiliations:** Department of Surgery, The Ohio State University Wexner Medical Center and James Comprehensive Cancer Center, Columbus, OH USA

**Keywords:** Outcomes, Cancer, Surgical oncologist, CGSO, Resection, Career stage

## Abstract

**Background:**

Practice patterns and potential quality differences among surgical oncology fellowship graduates relative to years of independent practice have not been defined.

**Methods:**

Medicare claims were used to identify patients who underwent esophagectomy, pancreatectomy, hepatectomy, or rectal resection for cancer between 2016 and 2021. Surgical oncology fellowship graduates were identified, and the association between years of independent practice, serious complications, and 90-day mortality was examined.

**Results:**

Overall, 11,746 cancer operations (pancreatectomy [61.2%], hepatectomy [19.5%], rectal resection [13.7%], esophagectomy [5.6%]) were performed by 676 surgical oncology fellowship graduates (females: 17.7%). The operations were performed for 4147 patients (35.3%) by early-career surgeons (1–7 years), for 4104 patients (34.9%) by mid-career surgeons (8–14 years), and for 3495 patients (29.8%) by late-career surgeons (>15 years). The patients who had surgery by early-career surgeons were treated more frequently at a Midwestern (24.9% vs. 14.2%) than at a Northeastern institution (20.6% vs. 26.9%) compared with individuals treated by late-career surgeons (*p* < 0.05). Surgical oncologists had comparable risk-adjusted serious complications and 90-day mortality rates irrespective of career stage (early career [13.0% and 7.2%], mid-career [12.6% and 6.3%], late career [12.8% and 6.5%], respectively; all *p* > 0.05). Surgeon case-specific volume independently predicted serious complications across all career stages (high vs. low volume: early career [odds ratio {OR}, 0.80; 95% confidence interval {CI}, 0.65–0.98]; mid-career [OR, 0.81; 95% CI, 0.66–0.99]; late career [OR, 0.78; 95% CI, 0.62–0.97]).

**Conclusion:**

Among surgical oncology fellowship graduates performing complex cancer surgery, rates of serious complications and 90-day mortality were comparable between the early-career and mid/late-career stages. Individual surgeon case-specific volume was strongly associated with postoperative outcomes irrespective of years of independent practice or career stage.

**Supplementary Information:**

The online version contains supplementary material available at 10.1245/s10434-024-15436-0.

Surgical oncology is a highly specialized field that combines the technical mastery of surgical interventions with the cognitive mastery of tumor biology, which forms the basis of oncologic decision-making.^1,2^ Surgical oncologists graduate from a general surgery residency program and pursue an additional 2-year training in Complex General Surgical Oncology (CGSO) before transitioning to independent practice.^2^ In the past decade, CGSO has become a recognized subspecialty by the Accreditation Council for Graduate Medical Education (ACGME), and since 2015, CGSO graduates are certified by the American Board of Surgery upon successfully completing training and passing the board exams.^1,3^

Transition to independent practice is a critical time in the development of new surgeons.^1,4,5^ After graduation from a fellowship, surgeons are expected to perform the operations themselves, assume responsibility for patient selection, and direct pre- and postoperative care.^1,4^ Surgical oncology fellowships were developed to help equip surgeons with the necessary skill set for independent practice in a field that involves technically challenging cancer operations.^1,2^ To date, there is a lack of information regarding practice patterns and outcomes of new surgical oncologists compared with more experienced surgeons. In fact, no prior study has assessed surgical outcomes of surgical oncology fellowship graduates in the United States across years of independent practice, mainly due to the lack of available data in any available administrative database. Whether surgical oncology fellowship programs adequately prepare surgeons for independent practice or not is thus, unknown. As such, the objective of the current study was to assess perioperative mortality and serious complications among a national representative cohort of Medicare beneficiaries undergoing complex cancer surgery by surgical oncology fellowship graduates across different career stages in the United States.

## Methods

### Data Sources and Study Cohort

Data were derived from 100% Medicare standard analytic files obtained by the Centers for Medicare and Medicaid Services (CMS) for fee-for-service beneficiaries enrolled in Medicare parts A and B. Patients 65 years of age or older who underwent pancreatectomy, hepatectomy, rectal resection, or esophagectomy for cancer between 2016 and 2021 were identified using International Classification of Diseases, 10th edition (ICD-10) revisions procedure codes. The study excluded patients not enrolled in Medicare parts A and B during the month of the surgical episode, individuals who received additional payments from a health maintenance organization, patients younger than 65 years, and patients who underwent surgery for benign diseases. For patients who underwent more than one surgical procedure, the first operation was considered the analytic procedure.

Surgeons were identified using the National Provider Identification (NPI) number associated with each procedure in the Medicare carrier file. The NPI numbers were manually screened by a review of publicly available sources including the American Board of Surgery (ABS) and institutional/hospital websites to select surgeons who had formally completed a surgical oncology fellowship (before ACGME accreditation) and CGSO (after ACGME accreditation). Surgeons were excluded if they had not completed a surgical oncology fellowship, had missing or inadequate information, or had completed other non-surgical oncology fellowships (including hepatopancreatobiliary [HPB] or transplant fellowships without having completed a surgical oncology fellowship). Patients who underwent cancer surgery by non-surgical oncologists were, in turn, excluded from the analytic cohort.

Information on the year of surgical oncology fellowship graduation was extracted and later linked to patient-level Medicare data. The American Hospital Association (AHA) database also was merged with patient-level data to incorporate hospital-level characteristics including teaching status, intensive care unit (ICU) availability, nurse-to-bed ratio, and number of beds. The final cohort of patients consisted of patients who underwent complex cancer surgery by fellowship-trained surgical oncologists.

### Variables of Interest and Outcomes

The variables of interest included patient characteristics (i.e., age, sex, race, social vulnerability index [SVI], Charlson score), procedural characteristics (i.e., type of procedure [pancreatectomy, hepatectomy, rectal resection, esophagectomy], year of surgery, admission type [elective vs. urgent]), hospital characteristics (i.e., location [Midwest, Northeast, South, West], rurality, teaching status, ICU availability, nurse-to-bed ratio, number of beds), and surgeon characteristics (i.e., surgeon sex and years of practice).

The primary independent variable was years of independent practice, calculated by subtracting the year the operation was performed from the year of surgical oncology fellowship graduation. To avoid outliers, surgeons in practice longer than 25 years were excluded due to the small sample size in each subsequent year. This resulted in a cohort of fellowship-trained surgical oncologists in independent practice between 1 and 25 years.

The primary outcomes were serious complications and 90-day mortality. Serious complications were defined as a complication coupled with extended length of stay (> 75th percentile).^6,7^ Postoperative complications were determined using previously validated ICD-10 clinical modification and procedure codes.^6,8,9^ Complications present at admission were excluded from the definition of severe complications, as previously described.^10^ Mortality was defined as death occurring within 90 days after the index operation.

The study was deemed exempt from approval by the institutional review board of the Ohio State University because the data were de-identified.

### Statistical Analysis

Descriptive statistics are presented as median values (interquartile range [IQR]) for continuous variables, and as frequency (%) for categorical variables. Differences in baseline characteristics were assessed with the Kruskal–Wallis one-way analysis of variance for continuous variables and with the chi-square test or Fisher’s exact test for categorical variables. The association of years of independent practice with serious complications and 90-day mortality was assessed by means of multivariable logistic regression analysis after adjustment for patient age, sex, race, SVI, Charlson score, year of surgery, type of surgery, admission type (elective vs. urgent), rurality, teaching status, nurse-to-bed ratio, number of beds, and surgeon sex. The cutoffs used to define early, mid, and late career stages were based on the tertiles of the total number of patients who underwent surgery by surgical oncologists in different years of individual practice.

A sensitivity analysis was performed to assess the association of surgeon case-specific volume (i.e., Leapfrog standards)^11^ with serious complications and 90-day mortality across different career stages of surgical oncologists. All tests were two-sided, and statistical significance was assessed at an alpha of 0.05. All analyses were performed using STATA, version 18.0 (StataCorp, College Station, TX, USA).

## Results

### Study Cohort

During the study period, 11,746 patients underwent complex cancer surgery (Table 1). The median patient age was 72 years (IQR, 68–77 years), and most of the patients were male (*n* = 6528, 55.6%) and white (*n* = 10,391, 88.5%). The majority of the patients underwent pancreatectomy (*n* = 7185, 61.2%) followed by hepatectomy (*n* = 2287, 19.5%), rectal resection (*n* = 1612, 13.7%), or esophagectomy (*n* = 662, 5.6%) for a malignant indication. A minority of the patients underwent cancer surgery in an urgent setting (*n* = 788, 6.7%). Most of the patients underwent cancer surgery at a teaching hospital (*n* = 8423, 71.7%) with ICU availability (*n* = 10,946, 99.6%) and more than 500 hospital beds (*n* = 8515, 72.8%) located in a metropolitan area (*n* = 9542, 81.5%).

**Table 1:** Characteristics of the study cohort

	Total (*n* = 11,746, 100%) *n* (%)	Treated by early-career SONC (*n* = 4147, 35.3%) *n* (%)	Treated by mid-career SONC (*n* = 4104, 34.9%) *n* (%)	Treated by late-career SONC (*n* = 3495, 29.8%) *n* (%)	*p* value
Age: years (IQR)	72 (68–77)	72 (68–77)	72 (68–77)	72 (68–77)	0.39
Patient sex					
Female	5218 (44.4)	1863 (44.9)	1825 (44.5)	1530 (43.8)	0.602
Male	6528 (55.6)	2284 (55.1)	2279 (55.5)	1965 (56.2)	
Race					
White	10,391 (88.5)	3649 (88.0)	3670 (89.4)	3072 (87.9)	0.057
Non-white	1355 (11.5)	498 (12.0)	434 (10.6)	423 (12.1)	
SVI					
Low	3930 (33.6)	1349 (32.6)	1398 (34.2)	1183 (34.0)	0.468
Medium	3949 (33.7)	1402 (33.9)	1387 (33.9)	1160 (33.3)	
High	3834 (32.7)	1388 (33.5)	1308 (32.0)	1138 (32.7)	
Charlson Score >4	4429 (37.7)	1643 (39.6)	1521 (37.1)	1265 (36.2)	0.005
Year of surgery					
2016	1820 (15.5)	620 (15.0)	693 (16.9)	507 (14.5)	0.056
2017	1972 (16.8)	682 (16.4)	706 (17.2)	584 (16.7)	
2018	1993 (17.0)	722 (17.4)	637 (15.5)	634 (18.1)	
2019	2024 (17.2)	724 (17.5)	697 (17.0)	603 (17.3)	
2020	1947 (16.6)	685 (16.5)	677 (16.5)	585 (16.7)	
2021	1990 (16.9)	714 (17.2)	694 (16.9)	582 (16.7)	
Procedure type					
Pancreatectomy	7185 (61.2)	2519 (60.7)	2540 (61.9)	2126 (60.8)	< 0.001
Hepatectomy	2287 (19.5)	839 (20.2)	831 (20.2)	617 (17.7)	
Rectal resection	1612 (13.7)	594 (14.3)	491 (12.0)	527 (15.1)	
Esophagectomy	662 (5.6)	195 (4.7)	242 (5.9)	225 (6.4)	
Admission type					
Elective	10,952 (93.3)	3864 (93.2)	3855 (94.0)	3233 (92.6)	0.041
Urgent	788 (6.7)	282 (6.8)	246 (6.0)	260 (7.4)	
Location					
Midwest	2549 (21.7)	1031 (24.9)	1021 (24.9)	497 (14.2)	< 0.001
Northeast	2615 (22.3)	856 (20.6)	818 (19.9)	941 (26.9)	
South	4950 (42.1)	1794 (43.3)	1664 (40.6)	1492 (42.7)	
West	1630 (13.9)	464 (11.2)	601 (14.6)	565 (16.2)	
Rurality					
Non-metropolitan	2171 (18.5)	836 (20.2)	732 (17.9)	603 (17.3)	0.002
Metropolitan	9542 (81.5)	3303 (79.8)	3361 (82.1)	2878 (82.7)	
Teaching status					
No	3323 (28.3)	1374 (33.1)	1011 (24.6)	938 (26.8)	< 0.001
Yes	8423 (71.7)	2773 (66.9)	3093 (75.4)	2557 (73.2)	
ICU availability	10,946 (99.6)	3897 (99.1)	3927 (99.9)	3122 (99.7)	< 0.001
Nurse-to-bed ratio (IQR)	2.2 (1.6–3.1)	2.2 (1.5–3.1)	2.2 (1.7–3.3)	2.1 (1.4–3.1)	< 0.001
Number of beds >500					
No	3182 (27.2)	1174 (28.4)	975 (23.8)	1033 (29.8)	< 0.001
Yes	8515 (72.8)	2956 (71.6)	3128 (76.2)	2431 (70.2)	
Surgeon sex					
Female	1331 (11.3)	707 (17.0)	486 (11.8)	138 (3.9)	< 0.001
Male	10,415 (88.7)	3440 (83.0)	3618 (88.2)	3357 (96.1)	
Years of experience (IQR)	10 (6–16)	4 (2–6)	11 (9–13)	19 (16–22)	< 0.001

SONC, surgical oncologist; IQR, interquartile range; SVI, social vulnerability index; ICU, intensive care unit

### Years of Experience Among Surgical Oncology Fellowship Graduates

**Fig. 1:**
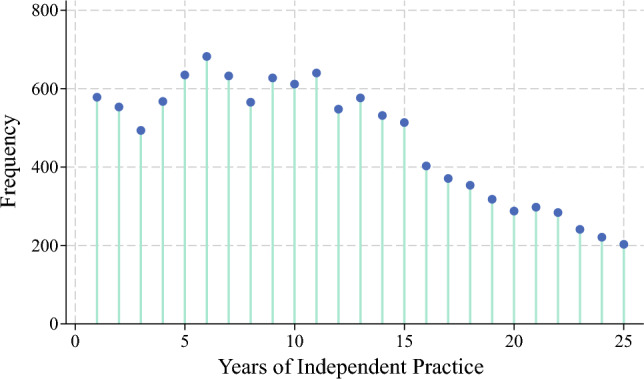
Lollipop chart showing the frequency of individuals who underwent complex cancer surgery relative to years of independent practice among surgical oncology fellowship graduates

During the study period, 676 surgeons (120 females [17.7%], 556 males [82.3%]) performed all the operations. The median years of independent practice among the surgical oncology fellowship graduates who managed all the cases was 10 years (IQR, 6–16 years; Fig. 1). Approximately one third of the patients (1st tertile: *n* = 4147, 35.3%) underwent surgery by a surgical oncologist in his or her 1st to 7th year of independent practice (early-career surgeons: *n* = 375, 55.5%). Another one third of the patients (2nd tertile: *n* = 4104, 34.9%) underwent surgery by a surgical oncologist in his or her 8th to 14th year of practice (mid-career surgeons: *n* = 155, 22.9%), whereas the remaining patients (3rd tertile; *n* = 3495, 29.8%) underwent surgery by a surgical oncologist in his or her 15th year of independent practice and beyond (late-career surgeons: *n* = 146, 21.6%).

The patients treated by early-career surgical oncologists more frequently had a Charlson score higher than 4 (39.6% vs. 36.2%) and underwent hepatectomy (20.2% vs. 17.7%) rather than esophagectomy (4.7% vs. 6.4%) in an elective setting (93.2% vs. 92.6%) compared with the individuals who underwent surgery by late-career surgical oncologists (all *p* < 0.05). The vast majority of the patients had their surgery performed by a male surgical oncologist (*n* = 10,415, 88.7%). The proportion of cases managed by female surgeons decreased across different career stages (early-career [17.0%] vs. mid-career [11.8%] vs. late-career [3.9%]; *p* < 0.001).

**Fig. 2:**
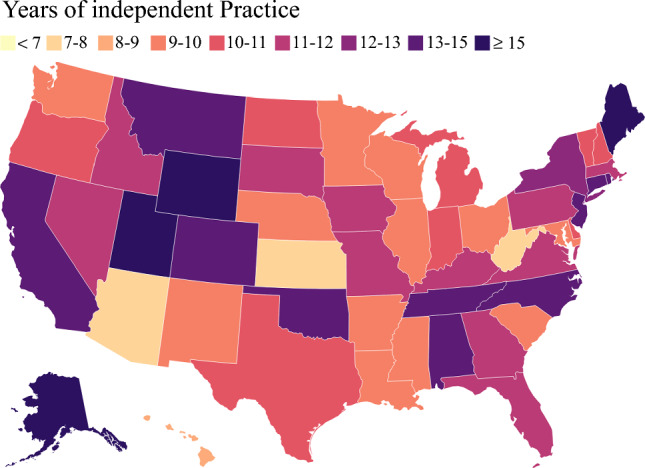
Distribution of cases relative to years of independent practice of surgical oncology fellowship graduates across the United States

The distribution of cases relative to years of independent practice of surgical oncology fellowship graduates across the United States varied considerably (Fig. 2). In particular, the patients whose surgery was performed by early-career surgeons less frequently had surgery in a metropolitan area (79.8% vs. 82.7%) and more frequently were treated at a Midwestern (24.9% vs. 14.2%) rather than a Northeastern (20.6% vs. 26.9%) institution compared with the individuals treated by late-career surgeons (all *p* < 0.05; Table 1). In addition, the patients treated by early-career surgeons less frequently underwent surgery for cancer at a teaching institution (66.9% vs. 73.2%) than the individuals treated by late-career surgeons (*p* < 0.05; Table 1).

### Serious Complications and 90-Day Mortality Across Different Career Stages

After complex cancer surgery, the overall incidence of serious complications was 12.8% (*n* = 1506), and the 90-day mortality was and 6.7% (*n* = 786). Serious complications and 90-day mortality varied markedly by procedure type. In particular, the incidence of 90-day mortality was higher after esophagectomy (9.5%) followed by hepatectomy (7.6%), pancreatectomy (6.5%), or rectal resection (5.2%) (*p* < 0.001). A similar trend was noted when serious complications were examined across different procedure types (esophagectomy [15.7%], hepatectomy [12.9%], pancreatectomy [12.6%], rectal resection [12.4%]; *p* = 0.138).

**Fig. 3:**
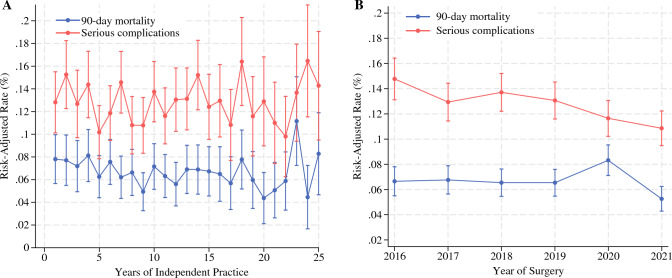
Risk-adjusted rates of serious complications and 90-day mortality after complex cancer surgery by **A** years of independent practice **B** and over time

After controlling for patient-, procedure-, hospital-, and surgeon-specific factors, surgical oncology fellowship graduates had comparable postoperative outcomes irrespective of whether they were early-, middle-, or late-career stage surgeons (early career: serious complications [13.0%] and 90-day mortality [7.2%]; mid career: serious complications [12.6%] and 90-day mortality [6.3%]; late career: serious complications [12.8%] and 90-day mortality [6.5%]; all *p* > 0.05; Fig. 3a). This was consistent across all the different procedure types except for a lower 90-day mortality after rectal resection performed by a late- versus early-career surgical oncologist (adjusted rate of 90-day mortality: 3.3% vs. 6.3%; odds ratio [OR], 0.48; 95% confidence interval [CI], 0.25–0.89; *p* = 0.039; Table 2). When trends in outcomes over time were assessed, the adjusted risk of serious complications decreased from 2016 to 2021 (14.8% vs. 10.9%; *p* < 0.001), yet the likelihood of 90-day mortality remained stable during the study period (2016 [6.6%] vs. 2021 [5.3%]; *p* = 0.57; Fig. 3b).

**Table 2:** Risk-adjusted rates (%) and OR (95% CI) of serious complications and 90-day mortality per procedure type and surgeon career stage

Risk-adjusted rates (%)	Early career% (IQR)	Mid career% (IQR)	Late career% (IQR)	*p* value*	OR (95% CI) (mid vs. early)	OR (95% CI) (late vs. early)
All procedures						
Serious complications	13.0 (12.0–14.0)	12.6 (11.6–13.6)	12.8 (11.7–13.9)	0.839	0.96 (0.84–1.10)	0.98 (0.85–1.13)
90-Day mortality	7.2 (6.4–8.0)	6.3 (5.6–7.1)	6.5 (5.7–7.3)	0.236	0.86 (0.72–1.03)	0.89 (0.74–1.07)
Pancreatectomy						
Serious complications	12.7 (11.4–14.0)	12.6 (11.3–13.9)	12.5 (11.1–13.9)	0.985	0.99 (0.84–1.18)	0.98 (0.82–1.18)
90-Day mortality	6.9 (5.9–7.9)	6.1 (5.2–7.1)	6.3 (5.3–7.4)	0.543	0.88 (0.70–1.11)	0.92 (0.72–1.16)
Hepatectomy						
Serious complications	12.4 (10.1–14.6)	13.3 (11.0–15.5)	13.0 (10.5–15.6)	0.860	1.08 (0.80–1.47)	1.06 (0.77–1.47)
90-Day mortality	7.8 (6.0–9.6)	7.0 (5.3–8.8)	8.3 (6.2–10.4)	0.654	0.89 (0.60–1.31)	1.07 (0.72–1.59)
Rectal resection						
Serious complications	12.3 (9.6–15.0)	11.8 (8.9–14.7)	13.1 (10.2–16.0)	0.815	0.95 (0.64–1.41)	1.08 (0.74–1.57)
90-Day mortality	6.3 (4.4–8.2)	6.3 (4.2–8.4)	3.3 (1.8–4.8)	**0.039**	1.01 (0.58–1.72)	**0.48 (0.25–0.89)**
Esophagectomy						
Serious complications	20.3 (14.7–25.9)	13.3 (8.9–17.6)	14.3 (9.5–19.1)	0.109	0.58 (0.34–1.01)	0.64 (0.37–1.12)
90-Day mortality	10.9 (6.5–15.4)	7.7 (4.3–11.1)	10.0 (6.1–13.9)	0.508	0.66 (0.32–1.36)	0.90 (0.44–1.82)

Bold values denote statistical significance

IQR, interquartile range; OR, odds ratio; CI, confidence interval

In addition, the outcomes for the patients who underwent a surgical procedure by surgeons completing a fellowship before versus after 2015 (eligible for ABS CGSO certification) were comparable (serious complications [OR, 1.11; 95% CI, 0.91–1.37], 90-day mortality [OR, 1.20; 95% CI, 0.91–1.59). Notably, most of the patients were treated by surgeons who had completed a fellowship at an ACGME-accredited fellowship program (*n* = 11,069, 94.3%). After adjustment for all relevant covariates, no differences in outcomes were noted between the patients treated by surgical oncologists who completed a fellowship at a currently ACGME-accredited institution and those who completed a fellowship at a non-accredited institution (serious complications [OR, 0.94; 95% CI, 0.74–1.19], 90-day mortality [OR, 1.39; 95% CI, 0.97–1.98).

### Impact of Surgeon Case-Specific Volume and Sex on Outcomes Across Different Career Stages

In the sensitivity analysis, individual surgeon case-specific volume (i.e., Leapfrog standards) was independently associated with lower odds of serious complications among individuals treated by surgical oncologists across all career stages (high- vs. low-volume: early career [OR, 0.80; 95% CI, 0.65–0.98], mid career [OR, 0.81; 95% CI, 0.66–0.99], late career [OR, 0.78; 95% CI, 0.62–0.97; Table 3). In contrast, surgeon case-specific volume was not associated with the incidence of 90-day mortality across all career stages. Rather, older patient age, higher Charlson score (patient-level factor), and lower nurse-to-bed ratio (hospital-level factor) remained independently associated with higher odds of 90-day mortality among patients treated by early-, middle-, and late-career surgical oncologists (Tables S1 and S2). Importantly, the odds of serious complications after complex cancer surgery were comparable between male and female surgical oncologists across all career stages (early career [OR, 1.02; 95% CI, 0.79–1.31], mid career [OR, 1.01; 95% CI, 0.75–1.35], late career [OR, 1.36; 95% CI, 0.77–2.40]). Similarly, the odds of 90-day mortality after complex cancer surgery were comparable irrespective of surgeon sex or career stage (male vs. female surgeon: early career [OR, 1.14; 95% CI, 0.81–1.60], mid career [OR, 1.27; 95% CI, 0.82–1.96], late career [OR, 2.54; 95% CI, 0.91–7.11]) (Tables S1 and S2).

**Table 3:** Sensitivity analysis examining the effect of individual surgeon case-specific volume with outcomes across different career stages

	Low surgeon volume	High surgeon volume	*p* value
Early-career surgeon			
*n* (%)^a^	2536 (61.1)	1611 (38.2)	
Serious complications^b^	Ref	0.80 (0.65–0.98)	**0.036**
90-Day mortality^b^	Ref	0.93 (0.71–1.21)	0.576
Mid-career surgeon			
*n* (%)^a^	1964 (47.9)	2140 (52.1)	
Serious complications^b^	Ref	0.81 (0.66–0.99)	**0.048**
90-Day mortality^b^	Ref	0.88 (0.67–1.16)	0.375
Late career surgeon			
*n* (%)^a^	1938 (55.4)	1557 (44.6)	
Serious complications^b^	Ref	0.78 (0.62–0.97)	**0.027**
90-Day mortality^b^	Ref	0.83 (0.61–1.12)	0.230

Bold *p*-values denote statistical significance

^a^Row percentages

^b^Values represent adjusted odds ratio (95% confidence interval)

## Discussion

The establishment of surgical oncology fellowships was driven by the growing complexity of cancer operations and the need for specialized training to provide high-quality, multidisciplinary care to cancer patients.^4^ Despite the well-established curriculum of surgical oncology fellowship training,^3^ no real-world assessment of outcomes exists among new surgical oncology fellowship graduates or their more experienced colleagues. The current study was important because we assessed outcomes of patients undergoing complex cancer surgery by surgical oncology fellowship graduates across different years of independent practice. Notably, surgical oncology fellowship graduates had comparable postoperative outcomes irrespective of whether they were early-career (1st to 7th year of independent practice), middle-career (8th to 14th year), or late-career (≥15th year) surgeons. This finding was consistent across all four surgical procedures examined (i.e., pancreatectomy, hepatectomy, rectal resection, and esophagectomy). Notably, an overall decrease in serious complications was noted in more recent years. In addition, individual surgeon case-specific volume (i.e., Leapfrog standards) was associated with lower odds of serious complications among surgical oncology fellowship graduates irrespective of years of independent practice. To the best of our knowledge, this is the first study to assess outcomes of surgical oncology fellowship graduates performing complex cancer surgery relative to years of independent practice in the United States.

Each year, more than 50 CGSO graduates enter the surgical oncology workforce.^1^ More than one-half of graduates obtain their first job as a faculty member at an academic facility.^12^ Large academic centers provide the infrastructure needed for complex cancer cases, including specialized intensive care units, subspecialty staff (i.e., advanced endoscopists, interventional radiologists, medical and radiation oncologists), while at the same time providing the environment and resources needed to conduct research.^12,13^

In terms of location, CGSO graduates are relatively spread out over the United States in a geographic distribution similar to that of their hometown and early training, with the majority concentrated in California, Texas, New York, Pennsylvania, and Florida.^12^ Building on previous research, the current study demonstrated a significant variation in the geographic dispersion of complex cancer cases managed by surgical oncology fellowship graduates according to years of independent practice. In particular, early-career surgical oncologists more frequently treated patients in the Midwest (24.9% vs. 14.2%) rather than the Northeast (20.6% vs. 26.9%) compared with late-career surgeons. In addition, early-career surgical oncologists less frequently treated patients in a teaching institution (66.9% vs. 73.2%) or an institution located in a metropolitan area (79.8% vs. 82.7%) than late-career surgeons.

The current study also demonstrated an under-representation of female surgeons (17.7% of all surgeons) performing complex cancer surgery in the United States. Importantly, most of the patients (*n* = 10,415, 88.7%) undergoing complex cancer surgery were treated by a male surgical oncologist during the study period. Perhaps more concerning was the finding that the proportion of cases managed by female surgeons decreased across different career stages (early career [17.0%] vs. mid career [11.8%] vs. late career [3.9%]; *p* < 0.001). This finding was particularly surprising given that the odds of serious complications and 90-day mortality after complex cancer surgery were comparable between male and female surgical oncologists across all the different career stages (Tables S1 and S2). These data were in line with those of previous studies reporting surgeon sex disparities in certain surgical subspecialties^14^ despite multiple reports highlighting equivalent outcomes between male and female surgeons.^15^ There is a notable under-representation of female surgeons, especially middle- and late-career female surgeons, performing complex cancer surgery. The data emphasize the need to support women in the field of surgical oncology and the importance of ongoing diversification of the surgical workforce. Further efforts should focus on determining the cause and mitigating the under-representation of female surgeons performing complex cancer surgery in the United States.

The fellowship curriculum of CGSO requires a minimum number of breast, endocrine, gastrointestinal, hepatopancreatobiliary, melanoma, and soft tissue cases.^1,2^ Despite the robustness of the CGSO fellowship curriculum,^3^ whether trainees are and feel adequately prepared for independent practice or not upon fellowship completion is not well understood. A recent survey of CGSO graduates from 2012 to 2022 demonstrated that 90% of graduates generally felt clinically prepared for independent practice, yet there were areas in which trainees felt unprepared both clinically and technically.^4^ Specifically, 43% of CGSO graduates felt clinically unprepared for thoracic operations, and 15% felt clinically unprepared for hepatobiliary operations.^4^ Similarly, 46% of CGSO graduates reported being technically unprepared for thoracic operations and 24% reported being technically unprepared for hepatobiliary operations upon fellowship completion.^4^

Building on prior research, the current study analyzed real-world data of patients who underwent complex cancer surgery by surgical oncology fellowship graduates in the United States. Given the lack of data available in administrative datasets, our team manually screened each individual surgeon record and identified surgeons who had formally completed a surgical oncology fellowship in the United States for further analysis. Notably, serious complications and 90-day mortality were comparable between newly graduated surgical oncologists and their more experienced colleagues, which was consistent across each examined surgical procedure. The only exception was lower 90-day mortality after rectal resection performed by late-career (3.3%) versus early-career (6.3%) surgeons. Given that the incidence of serious complications was comparable between early- and late-career surgeons, mortality was less likely to be related to the effect of the surgeon. Rather, patient-related factors (i.e., age and Charlson score) and hospital-level factors (i.e., nurse-to-bed ratio) were the main determinants of 90-day mortality after complex cancer surgery. These data were in line with those of previous studies reporting that patient-level factors, including advanced comorbidities, and increased age, contribute the most to an increased risk of mortality after complex surgical procedures.^16^

The current study also demonstrated that individual surgeon volume (i.e., Leapfrog standards) was an independent predictor of serious complications after complex cancer surgery performed by fellowship-trained surgical oncologists. Notably, a higher individual surgeon volume was associated with lower odds of serious complications, not only among newly graduated surgeons but also among middle-career (OR, 0.81; 95% CI, 0.65–0.98) and late-career (OR, 0.78; 95% CI, 0.62–0.97) surgical oncologists.

Although the volume-outcome in surgery is well-established,^17–19^ data from the current study demonstrated how surgical volume still affected outcomes of surgical oncologists performing complex cancer surgery independently of career stage. Collectively, although surgical oncology fellowship programs adequately and safely prepare surgeons’ transition into independent practice, clinical volume remained the driving factor determining postoperative outcomes. Specifically, individual surgeon volume rather than years of independent practice largely determined perioperative outcomes after complex cancer surgery. The findings highlight the need to maintain surgical volume, even among senior surgical oncologists, to ensure delivery of high-quality care to cancer patients.

One main strength of the current study was its use of the unique database built to capture all fellowship-trained surgical oncologists in the United States together with their information on year of fellowship graduation. Importantly, surgeon taxonomy codes, including both the NPPES file and CMS specialty codes, are frequently inaccurate.^20,21^ Therefore, previous studies have urged caution when these codes are used to describe surgeon sub-specialization.^20,21^ By manually searching each individual surgeon record, we were able to overcome this issue and provide the most accurate representation of all surgical oncologists performing complex cancer surgery in the United States to date.

Certain limitations, however, should be considered when the results of the current study are interpreted. Given the retrospective nature of the study, unmeasured confounding might exist that could have influenced the results. In addition, because the patient population was 65 years of age or older, the results might not be generalizable to younger populations or individuals not enrolled in Medicare. Furthermore, the availability of a senior partner during an early career may be important relative to patient outcomes. Due to the lack of this information in this dataset, we were unable to assess the association of senior partner availability with patient outcomes among early-career surgical oncologists. As with all billing databases, although ICD codes have a high fidelity of coding for surgical episodes, possible miscoding or variations in coding practices cannot be excluded. Nevertheless, Medicare provides the largest nationally representative sample of complex cancer surgery in the United States.

In conclusion, marked variation in the geographic dispersion of complex cancer cases managed by fellowship trained surgical oncologists according to years of independent practice was noted across the United States. Female surgical oncologists were largely under-represented in the surgical oncology workforce. Among surgical oncology fellowship graduates performing complex cancer surgery, the incidence of serious complications and 90-day mortality were comparable between their early and mid/late career stages. Individual surgeon case-specific volume was strongly associated with postoperative outcomes among surgical oncologists irrespective of years of independent practice and career stage. Although surgical oncology fellowship programs help prepare surgeons for independent practice, procedural volume remains an important driver of patient outcomes even among senior surgical oncologists.

### Supplementary Information

Below is the link to the electronic supplementary material.

Supplementary file1 (DOCX 22 kb)
