# Supplementary material for: Practice Patterns and Outcomes Among Surgical Oncology Fellowship Graduates Performing Complex Cancer Surgery in the United States Across Different Career Stages
Source: Ann Surg Oncol. 2024 May 18;31(8):4873–81. doi: 10.1245/s10434-024-15436-0 (PMC11236932; doi:10.1245/s10434-024-15436-0)
Supplement: Supplementary file 1 — Supplementary file1 (DOCX 22 kb) [file 10434_2024_15436_MOESM1_ESM.docx]

**Supplemental Table 1** Logistic regression examining odds of serious complications across different career stages

|  | **Early career** | | | **Middle career** | | | **Late career** | | |
| --- | --- | --- | --- | --- | --- | --- | --- | --- | --- |
| **Factors** | **OR** | **95%CI** | | **OR** | **95%CI** | | **OR** | **95%CI** | |
| **Age** | 1.01 | 1.00 | 1.03 | 1.03 | 1.01 | 1.05 | 1.01 | 1.00 | 1.03 |
| **Male (ref: female)** | 1.54 | 1.26 | 1.86 | 1.40 | 1.15 | 1.70 | 1.01 | 0.82 | 1.23 |
| **White (ref: non-White)** | 0.86 | 0.65 | 1.14 | 1.45 | 1.02 | 2.05 | 0.97 | 0.71 | 1.32 |
| **SVI (ref: low)** |  |  |  |  |  |  |  |  |  |
| **Medium** | 0.94 | 0.74 | 1.19 | 1.01 | 0.80 | 1.27 | 0.97 | 0.75 | 1.24 |
| **High** | 1.14 | 0.91 | 1.43 | 1.06 | 0.84 | 1.34 | 1.05 | 0.82 | 1.35 |
| **Charlson Score** | 1.03 | 1.01 | 1.06 | 1.06 | 1.03 | 1.09 | 1.05 | 1.02 | 1.09 |
| **Year of Surgery (ref: 2016)** |  |  |  |  |  |  |  |  |  |
| **2017** | 0.78 | 0.56 | 1.09 | 0.87 | 0.63 | 1.19 | 0.89 | 0.63 | 1.25 |
| **2018** | 0.93 | 0.68 | 1.27 | 1.07 | 0.78 | 1.47 | 0.84 | 0.60 | 1.18 |
| **2019** | 0.90 | 0.65 | 1.24 | 0.84 | 0.61 | 1.16 | 0.86 | 0.61 | 1.21 |
| **2020** | 0.78 | 0.56 | 1.08 | 0.79 | 0.57 | 1.09 | 0.71 | 0.50 | 1.01 |
| **2021** | 0.70 | 0.50 | 0.97 | 0.70 | 0.50 | 0.98 | 0.65 | 0.45 | 0.94 |
| **Type of Surgery (Ref: pancreatectomy)** |  |  |  |  |  |  |  |  |  |
| **Hepatectomy** | 0.92 | 0.70 | 1.18 | 1.11 | 0.87 | 1.41 | 1.10 | 0.84 | 1.45 |
| **Rectal resection** | 0.91 | 0.69 | 1.21 | 0.80 | 0.58 | 1.10 | 0.98 | 0.73 | 1.31 |
| **Esophagectomy** | 1.80 | 1.23 | 2.62 | 1.08 | 0.72 | 1.62 | 1.10 | 0.73 | 1.66 |
| **Urgent admission (ref: elective)** | 2.78 | 2.07 | 3.74 | 2.78 | 2.04 | 3.80 | 2.25 | 1.63 | 3.09 |
| **Metropolitan area (ref: non-metropolitan)** | 1.06 | 0.84 | 1.34 | 0.96 | 0.75 | 1.23 | 1.19 | 0.90 | 1.58 |
| **Teaching hospital (ref: non-teaching)** | 1.19 | 0.93 | 1.52 | 1.48 | 1.10 | 1.99 | 1.19 | 0.92 | 1.56 |
| **Nurse-to-bed ratio** | 1.04 | 0.97 | 1.11 | 1.14 | 0.96 | 1.22 | 0.98 | 0.92 | 1.06 |
| **Beds > 500** | 1.00 | 0.79 | 1.27 | 0.70 | 0.53 | 0.90 | 0.99 | 0.77 | 1.28 |
| **Male (ref: female surgeon)** | 1.02 | 0.79 | 1.31 | 1.01 | 0.75 | 1.35 | 1.36 | 0.77 | 2.40 |
| **High volume (ref: low volume) surgeon** | 0.80 | 0.65 | 0.98 | 0.81 | 0.66 | 0.99 | 0.78 | 0.62 | 0.97 |

**Supplemental Table 2** Logistic regression examining odds of 90-day mortality across different career stages

|  | **Early career** | | | **Middle career** | | | **Late career** | | |
| --- | --- | --- | --- | --- | --- | --- | --- | --- | --- |
| **Factors** | **OR** | **95%CI** | | **OR** | **95%CI** | | **OR** | **95%CI** | |
| **Age** | 1.04 | 1.02 | 1.06 | 1.08 | 1.06 | 1.10 | 1.06 | 1.03 | 1.08 |
| **Male (ref: female)** | 1.22 | 0.95 | 1.57 | 1.30 | 0.99 | 1.71 | 1.11 | 0.84 | 1.47 |
| **White (ref: non-White)** | 1.44 | 0.94 | 2.22 | 1.23 | 0.75 | 1.99 | 0.90 | 0.59 | 1.38 |
| **SVI (ref: low)** |  |  |  |  |  |  |  |  |  |
| **Medium** | 1.25 | 0.93 | 1.69 | 0.99 | 0.72 | 1.37 | 0.78 | 0.56 | 1.10 |
| **High** | 1.10 | 0.81 | 1.50 | 0.97 | 0.70 | 1.34 | 0.86 | 0.62 | 1.21 |
| **Charlson Score** | 1.11 | 1.07 | 1.16 | 1.11 | 1.06 | 1.15 | 1.09 | 1.04 | 1.14 |
| **Year of Surgery (ref: 2016)** |  |  |  |  |  |  |  |  |  |
| **2017** | 1.12 | 0.72 | 1.73 | 0.99 | 0.64 | 1.54 | 0.84 | 0.51 | 1.38 |
| **2018** | 0.98 | 0.63 | 1.52 | 0.90 | 0.57 | 1.43 | 1.03 | 0.65 | 1.66 |
| **2019** | 1.09 | 0.71 | 1.70 | 0.90 | 0.58 | 1.40 | 0.90 | 0.55 | 1.46 |
| **2020** | 1.36 | 0.90 | 2.08 | 1.15 | 0.75 | 1.77 | 1.25 | 0.79 | 1.98 |
| **2021** | 0.97 | 0.62 | 1.51 | 0.56 | 0.34 | 0.93 | 0.79 | 0.47 | 1.31 |
| **Type of Surgery (Ref: pancreatectomy)** |  |  |  |  |  |  |  |  |  |
| **Hepatectomy** | 1.16 | 0.86 | 1.58 | 1.26 | 0.91 | 1.76 | 1.45 | 1.03 | 2.05 |
| **Rectal resection** | 0.80 | 0.54 | 1.18 | 0.90 | 0.58 | 1.39 | 0.40 | 0.24 | 0.68 |
| **Esophagectomy** | 1.62 | 0.97 | 2.69 | 1.51 | 0.89 | 2.56 | 1.47 | 0.91 | 2.40 |
| **Urgent admission (ref: elective)** | 2.76 | 1.94 | 3.92 | 2.24 | 1.48 | 3.39 | 1.99 | 1.30 | 3.05 |
| **Metropolitan area (ref: non-metropolitan)** | 1.03 | 0.76 | 1.40 | 1.37 | 0.95 | 1.98 | 0.84 | 0.59 | 1.20 |
| **Teaching hospital (ref: non-teaching)** | 0.85 | 0.63 | 1.15 | 1.55 | 1.02 | 2.35 | 0.86 | 0.61 | 1.20 |
| **Nurse-to-bed ratio** | 0.83 | 0.75 | 0.92 | 0.91 | 0.82 | 0.99 | 0.77 | 0.69 | 0.86 |
| **Beds > 500** | 0.87 | 0.65 | 1.17 | 0.84 | 0.58 | 1.21 | 0.90 | 0.65 | 1.25 |
| **Male (ref: female surgeon)** | 1.14 | 0.81 | 1.60 | 1.27 | 0.82 | 1.96 | 2.54 | 0.91 | 7.11 |
| **High volume (ref: low volume) surgeon** | 0.93 | 0.71 | 1.21 | 0.88 | 0.67 | 1.16 | 0.83 | 0.61 | 1.12 |
